# Sorghum-based alcoholic beverage, *Burukutu,* perturbs the redox status of the liver of male rats

**DOI:** 10.1002/fsn3.139

**Published:** 2014-07-22

**Authors:** Taofeek O Ajiboye, Ganiyat A Iliasu, Oluwayemisi B Ojewuyi, Azeemat T Abdulazeez, Aisha O Muhammed, Fausat L Kolawole

**Affiliations:** 1 Antioxidants, Free Radicals, Functional Foods and Toxicology Research Laboratory, Department of Biological Sciences, Al-Hikmah UniversityIlorin, Nigeria; 2 Antioxidants, Free Radicals and Toxicology Research Laboratory, Biochemistry and Nutrition Unit, Department of Chemical Sciences, Fountain UniversityOsogbo, Nigeria; 3 Microbiology Unit, Department of Biological Sciences, Al-Hikmah UniversityIlorin, Nigeria; 4 Department of Home Economics and Food Science, University of IlorinIlorin, Nigeria

**Keywords:** *Burukutu*, DNA fragmentation, lipid peroxidation, protein oxidation, redox status, sorghum-based beer

## Abstract

The redox status of male rat liver following 28 days consumption of *Burukutu* was investigated. Twenty rats were randomized into four groups with five rats each. *Burukutu* consumption at 0.78 g/kg alcohol produced no significant change in the activities of alkaline phosphatase (ALP), alanine aminotransferase (ALT), and aspartate aminotransferase (AST). However, 3.71 and 7.43 g/kg dosages resulted in significant decrease in the activities of ALP, ALT and AST with corresponding increase in serum. The activity of cytochrome P_450_(CYP 2E1) increased significantly in the liver of rats following consumption of *Burukutu* at all doses investigated. The activities of superoxide dismutase, catalase, glutathione peroxidase, glutathione reductase and glucose 6-phosphate dehydrogenase decreased significantly (*P* < 0.05) in rats treat with 0.78 g/kg, 3.41 and 7.43 g/kg *Burukutu*. There was a significant increase in the level of glutathione disulfide (GSSG) with reduction in the levels of glutathione reduced (GSH) and GSH:GSSG. The levels of oxidative stress biomarkers, malondialdehyde, conjugated dienes, lipid hydroperoxides, protein carbonyl and percentage DNA fragmentation, increased significantly (*P* < 0.05). It is evident from the alterations in the activities of the hepatocellular enzymes, antioxidant enzymes and oxidative stress biomarkers that *Burukutu* mediated its toxicity through the depletion of the antioxidant enzymes.

## Introduction

Alcohol consumption is a central feature of adult (i.e., age 18 and older) life in Nigeria and plays a major role in social, religious, political, and economic relationships (Oshodin, 1995). Alcoholic beverages are consumed at virtually all ceremonies, including festivals, weddings, and funerals (Oshodin, 1995). Its consumption is a common practice in both rural and urban societies in Nigeria. Chronic alcohol consumption represents a major risk factor for the development of liver fibrosis, alcohol liver diseases (ALD), and hepatocellular carcinoma (HCC) (Hassan et al. 2002; Morgan et al. 2004; Pari and Karthikesan 2007). Alcohol-dependent induction of cytochrome P_450_ 2E1 (CYP2E1) leads to formation of acetaldehyde (Purohit et al. 2009). CYP2E1-dependent alcohol metabolism leads to increased hepatic oxidative stress due to the generation of reactive oxygen species (ROS) including hydroxyethyl radicals (McKillop and Schrum 2009). Studies have linked generation of ROS to ALD and HCC in many animals (Dupont et al. 1998; Gouillon et al. 2000; Morgan et al. 2002; Bradford et al. 2005). In Nigeria, due to poverty, locally brewed alcohol such as *burukutu* is widely consumed.

*Burukutu* is a popular alcoholic beverage of a vinegar-like flavour prepared from sorghum grains (Kolawole et al.*,* 2007). It is widely consumed as food (because it is thick and heavy) in the rural areas of northern Nigeria and in poor urban neighborhoods because it is more affordable than commercially brewed beer. The percentage alcohol content of *Burukutu* is between 3-6% (Bennett et al., 1998). *Burukutu* has been reported to contain vitamins, iron, magnesium, manganese, phosphorus, calcium, 26.7 g starch, and 5.9 g of protein per liter (Egemba and Etuk, 2007).

Despite arrays of studies done on the toxicological implications of alcohol, there is dearth information on the toxicological implication of *Burukutu*. This study thus investigates the effect of *Burukutu* consumption on the redox status of liver and some biomarkers of oxidative stress in rats.

## Materials and Methods

### Materials

#### Experimental animal

Two-month old, healthy male albino rats (*Rattus norvegicus*) of Wistar strain, weighing 183 ± 2.01 g were obtained from the Animal House of the Department of Veterinary Physiology, Biochemistry and Pharmacology, University of Ibadan, Nigeria. They were kept in clean plastic cages contained in well-ventilated house conditions with free access to feeds (Capfeed Ltd., Osogbo, Nigeria) and tap water. The animals were used according to the Guidelines of National Research Council Guide for the Care and Use of Laboratory Animals (National Research Council, 2011) and in accordance with the principles of Good Laboratory Procedure (GLP) [World Health Organization (WHO), 1998].

#### Chemicals and assay kits

Diphenylamine 5,5′-Dithio-bis(2-nitrobenzoic acid), guanidine hydrochloride, *N*-ethyl-maleimide (NEM), and salicylic acid, were procured from Research Organics, Cleveland, OH. Superoxide dismutase (SOD), glutathione peroxidase (GSH-Px), glutathione reductase (GSH-red) and glucose 6-phosphate dehydrogenase (Glc-6-PD) were products of Randox Laboratories Ltd., Co. Antrim, United Kingdom. All other reagents used were supplied by Sigma-Aldrich Inc., St. Louis, MO.

### Methods

#### Preparation of laboratory brewed *Burukutu*

*Burukutu* was prepared using the procedures described by Faparusi et al. (2007).

#### Animal treatment

Twenty (20) male rats were completely randomized into four groups (A–D) of five (5) animals each. Alcohol dosages of 0.78, 3.71 and 7.43 g/kg, which is equivalent to that consumed by light, moderate, and heavy drinkers was used in this study. Rats in groups B, C, and D were orally administered with *Burukutu* containing 0.78, 3.71, and 7.43 g/kg of alcohol, respectively, daily for 28 days. Group A, which served as the control was treated like the test groups except that the animals received distilled water. The animals were allowed free access to rat pellets and tap water. The animals were sacrificed 24 h after 28 days treatment.

#### Preparation of serum and tissue homogenates

The procedure described by Yakubu et al. (2009) and Ajiboye et al. (2014) was employed for the preparation of serum and tissue supernatants respectively.

#### Biochemical assay

The activities of alkaline phosphatase (ALP), alanine, and aspartate aminotransferases (AST) were determined as described by Wright et al. (1972) and Bergmeyer et al. (1986a,b), respectively. The activity of CYP2E1 was determined according to the procedure described by Dicker et al. (1990) SOD, Catalase, GSH-Px, GSH-red, and Glc 6-PD activities were assayed according to the procedures described by Beers and Sizer (1952) Mavis and Stellwagen (1968), Misra and Fridovich (1972), Rotruck et al. (1973), and DeMoss (1955), respectively. Levels of glutathione reduced and oxidized were assayed as described by Ellman (1959), and Hissin and Hilf (1976), respectively. The concentration of protein carbonyl in the liver homogenates was determined according to the procedure described by Levine et al. (1990). The concentrations of conjugated dienes, lipid hydroperoxides, and malondialdehyde were assessed according to the procedure described by Bus et al. (2001). The quantity of fragmented DNA was quantified according to the procedure described by Burton (1956).

### Statistical analysis

Results were expressed as the mean of five determinations ± SD. Analysis of variance (ANOVA) followed by the Tukey–Kramer test for differences between means was used to detect any significant differences (*P* < 0.05) between the treatment groups in this study using StatPlus, 2011 (AnalystSoft Inc., Alexandria, VA).

## Result and Discussion

**Figure 1:**
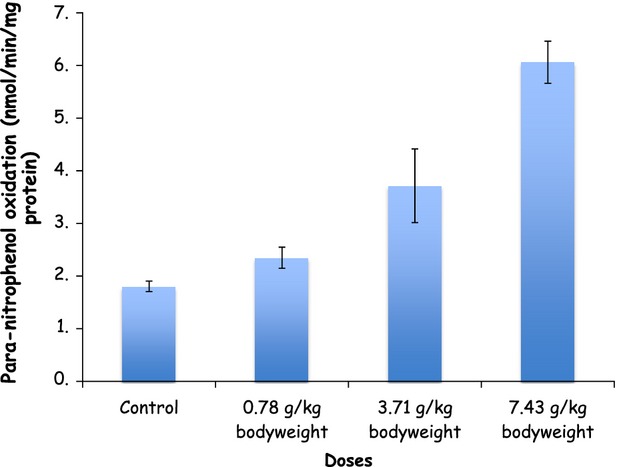
Specific activities of cytochrome P450 (2E1) in the liver of *Burukutu*-treated rats.

Chronic alcohol consumption has been reported to increase the CYP2E1 activity in the liver, resulting in increased ROS formation and eventually oxidative stress (Lu and Cederbaum 2008; McKillop and Schrum 2009). Although, 0.78 g/kg bodyweight of *Burukutu* produced no significant change (*P* > 0.05) in the activities of liver CYP2E1, 3.11 and 7.43 g/kg body weight produced a significant increase (*P* < 0.05) in the enzyme activities (Fig. 1). This increase may enhance ROS generation (superoxide anion, hydroxyl radicals, hydrogen peroxide, and hydroxyethyl radicals), which could lead to lipid peroxidation, oxidative protein damage, and DNA oxidation (Gouillon et al. 2000; Morgan et al. 2002; Bradford et al. 2005).

ALP, ALT, and AST are useful biomarkers of hepatic injury. Although, 0.78 g/kg bodyweight of *Burukutu* produced no significant (*P* > 0.05) change in the activities of ALP, ALT, and AST in the liver of male rats, these activities decreased significantly (*P* < 0.05) with corresponding increase in the serum following the administration of 3.11 and 7.43 g/kg body weight of *Burukutu* (Table 1). The alteration in ALP indicates plasma membrane labilization (Akanji et al. 1993). Also, alterations in ALT (cytosolic enzyme) and AST (mitochondrial enzymes) confirm that the plasma membrane integrity has been compromised, making the extracellular medium accessible by the cellular components such as ALT and AST.

**Table 1:** Specific activities of hepatocellular marker enzymes in rats following 28 days oral consumption of *Burukutu,* a sorghum-based alcoholic beverage.

Treatment/tissues	Alkaline phosphatase		Alanine aminotransferase		Aspartate aminotransferase	
	Liver	Serum	Liver	Serum	Liver	Serum
Control	7.25 ± 0.17^a^	0.034 ± 0.001^a^	72.05 ± 1.06^a^	2.48 ± 0.13^a^	104.17 ± 0.98^a^	5.64 ± 0.11^a^
0.78 g/kg bodyweight	7.25 ± 0.31^a^	0.046 ± 0.002^a^	74.25 ± 3.81^a^	2.23 ± 0.09^a^	107.27 ± 3.21^a^	6.03 ± 0.31^a^
3.71 g/kg bodyweight	4.63 ± 0.02^b^	0.192 ± 0.002^b^	48.12 ± 1.30^b^	4.46 ± 0.16^b^	88.24 ± 0.62^b^	10.14 ± 0.19^b^
7.43 g/kg bodyweight	2.33 ± 0.15^c^	0.547 ± 0.001^c^	21.85 ± 1.06^c^	10.61 ± 0.32^c^	61.11 ± 2.32^c^	23.18 ± 0.41^c^

Data are mean of five determinations ± SD. Specific enzyme activities are expressed as nmol min^−1^ mgprotein^−1^. Values carrying superscripts different for the liver and serum of each enzyme are significantly different (*P* < 0.05).

ROS production and oxidative stress is central to alcohol liver disease (Sergent et al. 2001; Das and Vasudevan 2007). During this condition, the activities of the antioxidant enzymes (SOD, CAT, GSH-Px, GSH-Red) defense arsenal are sometimes overwhelmed (Ajiboye 2010). The activities of SOD, CAT, GSH-Px, GSH-Red, Glc 6-PD significantly (P < 0.05) decreased in the liver of rats treated with *Burukutu* in a dose-dependent manner (Table 2). At the end of 28 days treatment, the highest dose of *Burukutu* used in this study produced 3.0 2.2, 2.9, 2.8, and 2.7 folds decrease in the activities of SOD, CAT, GSH-Px, GSH-Red, and Glc 6-PD, respectively. This decrease could predispose cellular macromolecules to oxidative rout of superoxide ion, hydroxyl radical, and hydrogen peroxide. Koch et al. (2004) reported a similar decrease in the activity of SOD following the repeated administration of ethanol.

**Table 2:** Specific activities of antioxidant enzymes in the liver of rats following 28 days oral consumption of *Burukutu,* a sorghum-based alcoholic beverage.

Treatments	Superoxide dismutase (nmol min^−1^ mgprotein^−1^)	Catalase (nmol min^−1^ mgprotein^−1^)	Glutathione peroxidase (nmol min^−1^ mgprotein^−1^)	Glutathione reductase(nmol min^−1^ mgprotein^−1^)	Glucose 6-phosphate dehydrogenase (nmol min^−1^ mgprotein^−1^)
Control	63.13 ± 3.21^a^	32.31 ± 3.38^a^	308.95 ± 2.22^a^	58.35 ± 0.79^a^	25.32 ± 1.30^a^
0.78 g/kg bodyweight	52.08 ± 1.31^b^	27.79 ± 1.29^b^	274.67 ± 4.34^b^	50.82 ± 0.13^b^	19.96 ± 0.81^b^
3.71 g/kg bodyweight	31.57 ± 2.09^c^	17.08 ± 1.12^c^	159.56 ± 2.70^c^	33.43 ± 1.12^c^	13.25 ± 0.14^c^
7.41 g/kg bodyweight	21.04 ± 1.20^d^	14.69 ± 1.32^c^	107.83 ± 0.23^d^	21.17 ± 0.42^d^	9.41 ± 0.31^d^

Data are mean of five determinations ± SD. Values carrying superscripts different for each parameter are significantly different (*P* < 0.05).

Nonenzymatic antioxidant system such as glutathione reduced (GSH) complements of the enzymatic antioxidants in the oxidative stress condition by acting as a free radical scavenger as well as modulating the functionality of the enzymes in vivo (Ajiboye et al. 2010). GSH homeostasis contributes to the toxic action of ethanol on the liver (Wu and Cederbaum 2005). Treatment of rats with *Burukutu* at all doses investigated resulted in a significant (*P* < 0.05) reduction in the levels of GSH and GSH:GSSG, and a significant (*P* < 0.05) elevation in the level of glutathione disulfide (GSSG) when compared to the control (Table 3). The significant loss of GSH might have resulted in the significant increase in GSSG. The decrease in GSH and increase in GSSG could lead to elevated mitochondrial levels of hydrogen peroxide and eventually hydroxyl radicals, which in turn may lead to lipid, protein, and DNA adduct formation, rendering the liver vulnerable to carcinogenesis (Purohit et al. 2013).Taylor et al. (2003) reported similar increases in the formation of GSSG and protein glutathionylation following the loss of GSH.

**Table 3:** Levels of nonenzymatic antioxidants in the liver of rats following 28 days oral consumption of *Burukutu,* a sorghum-based alcoholic beverage.

Treatments	Glutathione (reduced) (nmol mgprotein^−1^)	Glutathione (oxidized) (nmol mgprotein^−1^)	GSH:GSSG ratio
Control	52.18 ± 1.08^a^	3.23 ± 0.14^a^	16.15 ± 0.11^a^
0.78 g/kg bodyweight	47.50 ± 1.22^b^	5.21 ± 0.10^b^	9.12 ± 0.39^b^
3.71 g/kg bodyweight	34.23 ± 0.81^c^	12.68 ± 0.32^c^	2.70 ± 0.04^c^
7.41 g/kg bodyweight	20.48 ± 1.64^d^	21.93 ± 0.16^c^	0.97 ± 0.01^d^

Data are mean of five determinations ± SD. Values carrying superscripts different for each parameter are significantly different (*P* < 0.05).

Numerous studies have demonstrated the involvement of lipid peroxidation in alcohol-mediated toxicity (Shaw et al. 1988; Puddey and Croft 1997; Meagher et al. 1999). The levels of lipid peroxidation products; conjugated dienes, lipid hydroperoxides, and malonidialdehyde (Table 4) were significantly (*P* < 0.05) elevated in the liver of rats treated with *Burukutu* in all the doses investigated (Table 4). These elevations indicate toxicity and oxidative stress.

**Table 4:** Levels of lipid peroxidation products in the liver of rats following 28 days oral consumption of *Burukutu,* a sorghum-based alcoholic beverage.

Treatments	Conjugated dienes (nmol mgprotein^−1^)	Lipid hydroperoxides (nmol mgprotein^−1^)	Malondialdehyde (nmol mgprotein^−1^)
Control	35.16 ± 0.62^a^	23.43 ± 0.25^a^	5.32 ± 0.13^a^
0.78 g/kg bodyweight	39.98 ± 1.02^b^	28.02 ± 0.41^b^	8.14 ± 0.35^b^
3.71 g/kg bodyweight	52.46 ± 2.31^c^	43.46 ± 1.20^c^	15.62 ± 0.18^a^
7.41 g/kg bodyweight	67.94 ± 1.19^c^	53.41 ± 1.25^d^	21.45 ± 0.21^c^

Data are mean of five determinations ± SD. Values carrying superscripts different for each parameter are significantly different (*P* < 0.05).

Oxidative damage to cellular protein is one of the deleterious outcomes of chronic ethanol consumption (Abraham et al. 2002). Also, acute and chronic alcohol exposure has been shown to damage DNA in a variety of systems, cells, and species, including humans (Wu and Cederbaum 2003). The level of protein carbonyl increased significantly (*P* < 0.05) in the liver of *Burukutu*-treated rats (Table 5). In a similar vein, there was a significant (*P* < 0.05) increase in the fragmented DNA in the liver of rats treated with *Burukutu* (Table 5). This could lead to irreversible loss of protein function and play a role in experimental ALD (Fataccioli et al. 1999). The significant increase in the percentage of fragmented DNA in *Burukutu-*treated indicates genotoxicity.

**Table 5:** Levels of protein carbonyl and fragmented DNA in the liver of rats following 28 days oral consumption of *Burukutu,* a sorghum-based alcoholic beverage.

Treatments	Protein carbonyl (nmol mgprotein^−1^)	Fragmented DNA (%)
Control	3.20 ± 0.13^a^	4.72 ± 0.45^a^
0.78 g/kg bodyweight	4.75 ± 0.16^b^	6.62 ± 1.51^b^
3.71 g/kg bodyweight	6.36 ± 0.05^a^	25.00 ± 0.11^c^
7.41 g/kg bodyweight	9.06 ± 0.01^b^	57.32 ± 2.16^c^

Data are mean of five determinations ± SD. Values carrying superscripts different for each parameter are significantly different (*P* < 0.05).

## Conclusion

Findings from this study show that sorghum-based alcoholic beverage, *Burukutu,* perturbed redox status of rats. This could have resulted from the appreciable amount of alcohol in the beverage, whose metabolism results in the generation of ROS that causes oxidative perturbation of cellular macromolecules. Thus indiscriminate consumption of the beverage should be avoided as it could cause detrimental effect on health and well-being.
